# Prevalence of articular disc displacement among Thai TMD patients: a retrospective study on the association with demographic and clinical characteristics

**DOI:** 10.22514/jofph.2026.028

**Published:** 2026-03-12

**Authors:** Uthai Uma, Wacharasak Tumrasvin

**Affiliations:** ^1^Department of Occlusion, Faculty of Dentistry, Chulalongkorn University, 10330 Bangkok, Thailand; ^2^Department of Prosthodontics, Faculty of Dentistry, Chulalongkorn University, 10330 Bangkok, Thailand

**Keywords:** Disc displacement, Temporomandibular joint, Occlusion, Risk factors, Mouth opening, Oral habits, TMD

## Abstract

**Background**: Disc displacement (DD) is among the most prevalent intra-articular temporomandibular disorders. Identifying associated factors can support early diagnosis and management. The objective of this study was to evaluate the relationship between disc conditions and patient demographics and clinical characteristics. **Methods**: This retrospective study analyzed 770 patient records collected from 2021 to 2025 using data extracted from the hospital’s digital system. Variables included demographic information, behavioral habits, occlusal characteristics, clinical findings, and temporomandibular joint (TMJ) diagnoses. Statistical analyses were performed using descriptive statistics, chi-square tests, independent *t*-tests, one-way analysis of variance, and binary logistic regression, with significance set at *p *< 0.05. **Results**: DD was diagnosed with 420 patients (54.5%). DD patients were significantly younger (mean 39.0 years, *p *< 0.001), predominantly in the 21–40 age group (*p *= 0.002), and more often female (72.0%, *p *< 0.001). Behavioral habits such as resting the chin on the hand (*p * < 0.001) and previous orthodontic treatment (*p* = 0.010) were more prevalent in the DD group. Occlusal characteristics, including overjet, overbite, midline deviation, and occlusal scheme, showed no significant association with DD. However, DD patients exhibited reduced posterior and total static articulation (*p *< 0.05), as well as decreased working contacts and increased non-working contacts during the right excursion. The distribution of TMJ clicking and disc diagnoses was comparable between the left and right sides. More advanced subtypes of DD were linked to younger age, female sex, reduced mouth opening capacity, and greater mandibular deviation. **Conclusions**: DD was associated with demographic and behavioral factors, particularly younger age, female sex, and certain oral habits. Functional occlusal contacts were also found to be associated with an increased likelihood of DD. Comprehensive assessment is essential for diagnosis and management.

## 1. Introduction

Temporomandibular disorders (TMD) comprise a heterogeneous group of conditions affecting the temporomandibular joint (TMJ), masticatory muscles, and associated structures involved in mandibular function [1]. These disorders are characterized by a multifactorial pathophysiology, encompassing anatomical, functional, psychological, and behavioral components [1, 2]. The global prevalence of TMD is estimated at approximately 34% [3], with an incidence of 6.1% reported among Thai patients [4].

Within the spectrum of TMDs, disc displacement (DD) is one of the most frequently observed intra-articular disorders, ranging from 18%–35% [5]. This condition involves an abnormal positional relationship between the mandibular condyle and the articular disc within the TMJ [6]. The Diagnostic Criteria for Temporomandibular Disorders (DC/TMD) provides a standardized framework for classifying these conditions based on patient’s history and clinical examination [7]. DD manifests as either disc displacement with reduction (DDwR), often accompanied by joint sounds, such as clicking or popping [8], or disc displacement without reduction (DDwoR), typically associated with restricted movement or locking [9].

DDs are believed to be influenced by various factors, including occlusal parameters such as anterior guidance, overjet, overbite, and both static and dynamic occlusal contacts [10, 11]. These disorders represent one of the primary causes of orofacial pain and functional impairment, typically presenting with symptoms such as joint sounds, pain during mandibular movement, and limited mouth opening [5]. However, the complex and multifactorial nature of these disorders makes it difficult to establish definitive causal relationships. Understanding the associations between demographic or clinical characteristics and DD is critical for enhancing diagnostic accuracy and developing individualized treatment strategies [12].

Despite extensive investigations, consensus remains lacking regarding the precise relationship between DD and demographic or clinical characteristics. Previous studies have reported conflicting results, with some identifying significant associations while others find limited or no correlation [13, 14]. Moreover, many earlier investigations relied on subjective or simplified assessments of occlusion, such as malocclusion classification or the presence of balancing-side contacts, rather than detailed, quantitative analysis of occlusal contact distribution during mandibular movements [13, 14, 15, 16]. One such quantitative metric, the articulation ratio, which measures the distribution of occlusal contacts across the intercuspal position (ICP), right excursion (RE), left excursion (LE), and protrusion (PRO), has been largely underutilized in TMD research.

Therefore, this retrospective study aimed to examine the association between demographic and clinical characteristics and articular DD among Thai patients seeking care at the Occlusion and Orofacial Pain Clinic, Chulalongkorn Dental Hospital. By analyzing comprehensive digital medical records collected over a five-year period (2021–2025), the study seeks to identify significant factors associated with different types of DD as categorized by the DC/TMD framework. The findings are intended to provide clinically relevant insights into the diagnosis and management of TMDs and support the development of more targeted, evidence-based treatment protocols. The null hypothesis of this study was that there was no significant association between demographic or clinical characteristics and the presence or type of articular DD in Thai patients diagnosed with TMD.

## 2. Materials and methods

### 2.1 Samples

This research employed a retrospective approach, gathering data from medical charts stored in the digital system. Ethical clearance was obtained from the Human Research Ethics Committee of the Faculty of Dentistry, Chulalongkorn University, Bangkok, Thailand (HREC-DCU 2024-107). All records in the digital system were supervised by the Dean of the Faculty of Dentistry, who held authority over all stages of the research, including activities before, during, and after data collection. The requirement for individual informed consent was waived by the Human Research Ethics Committee; however, the Dean provided consent for access to patient data. The ethical approval was valid from 13 December 2024 to 12 December 2026. Data collection was conducted from March 2025 to June 2025, covering retrospective records from September 2021 to May 2025.

The samples in this study were required to meet the following inclusion criteria (Fig. 1): (1) patients must have received treatment at the Occlusion and Orofacial Pain Clinic, Chulalongkorn Dental Hospital, between September 2021 and May 2025; (2) must have undergone both intraoral and extraoral examinations with corresponding records documented in the digital medical system; and (3) must have had at least 20 natural teeth and not be wearing any removable dentures. Once the target medical charts were identified, they were excluded if they met any of the following criteria: (1) incomplete documentation, or (2) missing data relevant to the study objectives.

**Fig. 1.** Flow diagram of screening medical records.

### 2.2 Sample size

The study investigated the prevalence of DD among Thai patients seeking treatment at the Occlusion and Orofacial Pain Clinic. The participating patients were divided into two subgroups: those with normal discs and those with disc abnormalities. As a result, the sample size was calculated separately for each group. Due to the large and undefined nature of the patient population, Cochran’s formula was applied to determine the appropriate sample size [17]. The formula used was n = [*Z*^2^(P(1 − P))]/E^2^, where *Z* was 1.96 for a 95% confidence level, the expected proportion (P) was 0.5, and the margin of error (E) was 5%. This calculation yielded a required sample size of 385 medical records per subgroup, resulting in a total of 770 medical charts for the study.

### 2.3 Research instruments

A structured data collection form was developed as the primary research instrument to guide systematic data gathering for the study. It consisted of four main sections:

The first section captured demographic information, including hospital number (HN), age (in years), age group, sex (female/male), history of previous orthodontic treatment (yes/no), and behavioral habits such as unilateral chewing (yes/no), work-related poor posture (yes/no), sleeping on one side (yes/no), and resting the chin on the hand (yes/no).

The second section focused on clinical examination findings. It included the number of remaining teeth, measurements of overjet (mm), overbite (mm), midline deviation (mm), and slide in centric (mm). Morphologic classifications of anterior and posterior malocclusion were also recorded. Patterns of mouth opening were categorized as straight (no deviation from the midline), corrected deviation (≤2 mm), or uncorrected deviation (>2 mm). Additionally, maximum mouth opening (in mm) was measured under three conditions: pain-free, unassisted (without the examiner’s assistance), and assisted (with the examiner’s assistance).

The third section involved the occlusal factors. Medical records of occlusal contacts were obtained using a standard form routinely employed in the clinic, which included assessments of occlusion in ICP, RE, LE, and PRO. Measurements were taken with the patient in a supine position after gently drying the oral cavity with an air spray. Each measurement was performed twice, first by dental students and then verified by calibrated academic staff specializing in Occlusion and Orofacial Pain, using shim stock (8 μm), following the standard protocol incorporated into the curriculum. This section analyzed data from occlusal contacts originally recorded in the clinic, which were subsequently used to evaluate occlusal schemes and calculate occlusal contacts. Occlusal schemes on the working sides were categorized according to the Glossary of Prosthodontic Terms, 10th edition (2023) [18], as anterior guidance, posterior guidance, canine guidance, group function, balanced occlusion, unclassified (not fitting any other category), and no guidance (absence of working guidance with presence of non-working interference). Occlusal contacts were assessed by determining the number of lower teeth with positive occlusal contacts relative to the total number of remaining lower teeth. This criterion, formulated by the authors [19], was utilized to calculate articulation ratios for both static and dynamic mandibular positions, as presented in Table 1.

**Table 1.** Articulation ratio and corresponding formula used to calculate occlusal contact in occlusal analysis.

Mandibular Position	Articulation Type	Articulation Formula
Intercuspal Position (ICP)		
	Anterior Static Articulation (ASA)	ASA = A/Ta
	Posterior Static Articulation (PSA)	PSA = P/Tp
	Total Static Articulation (TSA)	TSA = (A + P)/(Ta + Tp)
Right Excursion (RE), Left Excursion (LE)		
	Working Lateral Articulation (WLA)	WLA = W/Tw
	Non-working Lateral Articulation (NLA)	NLA = N/Tn
	Total Lateral Articulation (TLA)	TLA = (W + N)/(Tw + Tn)
Protrusion (PRO)		
	Anterior Protrusive Articulation (APA)	APA = A/Ta
	Posterior Protrusive Articulation (PPA)	PPA = P/Tp
	Total Protrusive Articulation (TPA)	TPA = (A + P)/(Ta + Tp)

*Number of lower positive tooth contacts: A, Anterior; P, Posterior; W, Working side; N, Non-working side. Total number of remaining lower teeth: Ta, Anterior; Tp, Posterior; Tw, Working side; Tn, Non-working side.*

The fourth section included clinical signs and diagnostic information. This involved the presence of TMJ clicking (yes/no), clicking types (opening, closing, or eccentric clickings). Diagnostic categories for articular disc disorders were documented according to the Diagnostic Criteria for Temporomandibular Disorders (DC/TMD) [7], including: disc displacement with reduction (DDwR), disc displacement with reduction with intermittent locking (DDwRwIL), disc displacement without reduction with limited opening (DDwoRwLO), and disc displacement without reduction without limited opening (DDwoRwoLO).

### 2.4 Data collection

An initial version of the data collection form was developed and tested in a pilot study. Based on feedback and preliminary findings, the authors revised the form to produce the final version. To ensure consistency, only one data collector was assigned to gather patient data; therefore, only intra-rater reliability was assessed, rather than inter-rater reliability. The finalized form was evaluated through a test–retest procedure with a one-month interval. The assessment of intra-rater reliability, conducted on a pilot group of 100 patients, yielded an intraclass correlation coefficient (ICC) of 0.999, indicating almost perfect agreement. Subsequently, all items were digitized and transferred to an online platform using Google Forms. The designated data collector accessed patient records from the digital database spanning September 2021 to May 2025. Only data relevant to the study were extracted according to predefined guidelines. Each data point was collected independently and organized for subsequent statistical analysis.

### 2.5 Statistical analysis

Statistical analyses were performed using SPSS version 29.0 (IBM Corp., Armonk, NY, USA). Descriptive statistics included means, standard deviations (SD), frequencies (N), and percentages (%). For comparative analyses, the Chi-square and compare proportions tests were employed to assess differences in categorical variables. Binary logistic regression was also applied to evaluate associations between categorical variables and DD. The normality of continuous data was evaluated using the Kolmogorov-Smirnov test. Data that met normality assumptions were compared using independent *t*-tests and one-way analysis of variance (ANOVA), followed by Tukey’s *post hoc* test for multiple comparisons. A *p*-value of less than 0.05 was considered statistically significant in all analyses.

## 3. Results

The study reviewed medical charts from a total of 790 patients who met all inclusion criteria (Fig. 1). Of these, 20 patients were excluded due to incomplete documentation. The final sample consisted of 770 patients, whose data were analyzed to explore associations between various demographic and behavioral factors and articular DD. Among the included cases, 420 patients (54.5%) were diagnosed with DD.

As shown in Table 2, individuals with DD were significantly younger than those with normal discs (mean age: 39.0 ± 16.1 years *vs*. 42.7 ± 16.7 years, *p * < 0.001), and age distribution differed significantly (*p* = 0.002), with DD more prevalent in the 21–40 age group (49.9%). DD was also significantly more common among females (72.0%, *p * < 0.001). Additionally, a history of orthodontic treatment (*p* = 0.010) and the habit of resting the chin on the hand (*p * < 0.001) were more frequently observed in DD patients. In contrast, no significant associations were found for unilateral chewing, work-related poor posture, or sleeping on one side. These findings suggest that articular DD are associated with certain demographic and behavioral factors—particularly sex, age, orthodontic history, and chin-resting habits.

**Table 2.** Comparison of demographic and behavioral risk factors between patients with normal disc position and disc displacement.

Variables		Normal Disc (N = 350)	Disc Displacement (N = 420)	Total (N = 770)	Crude OR [95% CI]	*p*-value	Adjusted OR [95% CI]	*p*-value
Age^¶^ (yr)								
	Mean ± SD	42.7 ± 16.7	39.0 ± 16.1	40.7 ± 16.5	N/A	<0.001*	N/A	<0.001*
	[95% CI]	[41.0–44.5]	[37.4–40.5]	[39.5–41.8]				
Age groups^†^ (yr)								
	0–20	30 (8.6%)	37 (8.8%)	67 (8.7%)	N/A	0.002*	N/A	0.026*
	21–40	129 (37.0%)^a^	210 (49.9%)^b^	339 (44.0%)				
	41–60	131 (37.5%)^a^	114 (27.1%)^b^	245 (31.8%)				
	61–80	60 (17.1%)	59 (14.0%)	119 (15.5%)				
Sex^†^								
	Female	208 (59.6%)^a^	303 (72.0%)^b^	511 (66.4%)	1.77 [1.31–2.39]	<0.001*	1.78 [1.30–2.44]	<0.001*
	Male	142 (40.6%)^a^	117 (27.9%)^b^	259 (33.6%)				
Previous orthodontic treatment^†^								
	Yes	71 (20.3%)^a^	119 (28.3%)^b^	190 (24.7%)	1.55 [1.11–2.17]	0.010*	1.25 [0.88–1.79]	0.220
	No	279 (79.7%)^a^	301 (71.7%)^b^	580 (75.3%)				
Unilateral chewing^†^								
	Yes	149 (42.6%)	188 (44.7%)	336 (43.6%)	1.08 [0.81–1.44]	0.586	1.00 [0.74–1.35]	0.999
	No	201 (57.4%)	233 (55.3%)	434 (56.4%)				
Work-related poor posture^†^								
	Yes	34 (9.7%)	55 (13.1%)	89 (11.6%)	1.40 [0.89–2.20]	0.144	1.23 [0.77–1.98]	0.391
	No	316 (90.3%)	365 (86.9%)	681 (88.4%)				
Sleeping on one side^†^								
	Yes	158 (45.1%)	198 (47.1%)	356 (46.2%)	1.08 [0.82–1.44]	0.579	0.85 [0.62–1.16]	0.302
	No	192 (54.9%)	222 (52.9%)	414 (53.8%)				
Resting chin on hand^†^								
	Yes	73 (20.9%)^a^	134 (31.9%)^b^	207 (26.9%)	1.78 [1.28–2.47]	<0.001*	1.54 [1.06–2.23]	0.023*
	No	277 (79.1%)^a^	286 (68.1%)^b^	563 (73.1%)				

OR, Odds Ratio; SD, Standard Deviation; CI, Confidence Interval; N/A, Not Applicable. ^¶^independent t-test; ^†^Chi-square test for crude OR and binary logistic regression test for adjusted OR. ^a,b^statistical significance from the compare proportions test. *statistical significance (p < 0.05).

As seen in Table 3, the study examined occlusal characteristics and the range of motion concerning DD among 770 patients. There were no significant differences between groups in the number of remaining teeth, overjet, overbite, midline deviation, slide in centric, or anterior and posterior morphologic malocclusions (all *p * > 0.05), suggesting that the presence of DD is not strongly associated with general dental alignment and occlusal relationships.

**Table 3.** Comparison of occlusal characteristics, morphologic malocclusion, and occlusal scheme between patients with normal disc position and disc displacement.

Variables		Normal Disc (N = 350)	Disc Displacement (N = 420)	Total (N = 770)	*p*-value
Remaining teeth^¶^ (teeth)					
	Mean ± SD	27.2 ± 2.3	27.3 ± 2.2	27.3 ± 2.2	0.357
	[95% CI]	[27.0–27.4]	[27.1–27.5]	[27.1–27.4]	
Overjet^†^					
	Large (≥3 mm)	192 (54.9%)	221 (52.6%)	413 (53.6%)	0.535
	Normal (<3 mm)	158 (45.1%)	199 (47.4%)	357 (46.4%)	
Overbite^†^					
	Large (≥3 mm)	174 (49.7%)	203 (48.3%)	377 (49.0%)	0.703
	Normal (<3 mm)	176 (50.3%)	217 (51.7%)	393 (51.0%)	
Midline deviation^†^					
	Yes	238 (68.0%)	282 (67.1%)	520 (67.5%)	0.800
	No	112 (32.0%)	138 (32.9%)	250 (32.5%)	
Slide in centric^†^					
	Large (≥2.0 mm)	51 (14.6%)	70 (16.7%)	121 (15.7%)	0.725
	Small (0.5–1.5 mm)	252 (72.0%)	296 (70.5%)	548 (71.2%)	
	No (0 mm)	47 (13.4%)	54 (12.9%)	101 (13.1%)	
Anterior morphologic malocclusion^†^					
	Normal	106 (30.3%)	140 (33.3%)	246 (31.9%)	0.593
	Deep bite	169 (48.3%)	198 (47.1%)	367 (47.7%)	
	Edge-to-edge	55 (15.7%)	52 (12.4%)	107 (13.9%)	
	Open bite	14 (4.0%)	20 (4.8%)	34 (4.4%)	
	Cross bite	6 (1.7%)	10 (2.4%)	16 (2.1%)	
Posterior morphologic malocclusion^†^					
	Normal	299 (85.4%)	352 (83.8%)	651 (84.5%)	0.761
	Cross bite	37 (10.6%)	47 (11.2%)	84 (10.9%)	
	Scissor bite	14 (4.0%)	21 (5.0%)	35 (4.5%)	
Right excursion^†^ (working side)					
	Anterior guidance	44 (12.6%)	49 (11.6%)	94 (12.2%)	0.156
	Canine guidance	50 (14.3%)	60 (14.3%)	110 (14.3%)	
	Group function	129 (36.9%)	146 (34.7%)	275 (35.7%)	
	Balanced occlusion	66 (18.9%)^a^	108 (25.7%)^b^	173 (22.5%)	
	Unclassified	47 (13.4%)^a^	38 (9.0%)^b^	84 (10.9%)	
	No guidance	14 (4.0%)	20 (4.8%)	34 (4.4%)	
Left excursion^†^ (working side)					
	Anterior guidance	48 (13.7%)	49 (11.7%)	97 (12.6%)	0.354
	Canine guidance	55 (15.7%)	61 (14.5%)	116 (15.1%)	
	Group function	112 (32.0%)	144 (34.3%)	256 (33.2%)	
	Balanced occlusion	80 (22.9%)	101 (24.0%)	181 (23.5%)	
	Unclassified	42 (12.0%)	38 (9.0%)	80 (10.4%)	
	No guidance	13 (3.7%)	27 (6.4%)	40 (5.2%)	
Protrusion^†^					
	Anterior guidance	205 (58.6%)	240 (57.1%)	445 (57.8%)	0.908
	Balanced occlusion	71 (20.3%)	90 (21.4%)	161 (20.9%)	
	Posterior guidance	74 (21.1%)	90 (21.4%)	164 (21.3%)	

*SD, Standard Deviation; CI, Confidence Interval. ^¶^independent t-test, ^†^Chi-square test, ^a,b^statistical significance from the compare proportions test.*

The study also evaluated the relationship between occlusal scheme and DD, as shown in Table 3. No significant association was found between the types of occlusal scheme and DD across all mandibular movements (RE, LE, and PRO) (*p * > 0.05). These findings indicate no significant differences in general occlusal scheme types between patients with and without DD. However, when analyzed using the compare proportions test for the RE, the prevalence of balanced occlusion was significantly higher in patients with DD compared with those with normal disc position.

The calculated occlusal contacts are illustrated in Fig. 2. Significant differences were observed in several occlusal contact parameters. In ICP, the DD group demonstrated lower values in posterior static articulation (0.822, *p* = 0.028) and total static articulation (0.613, *p* = 0.019), suggesting reduced stability in posterior and overall static contacts. Additionally, RE measurements revealed notable differences: individuals with DD showed lower working guidance (0.286 *vs*. 0.316, *p* = 0.015) and higher non-working guidance (0.056 *vs*. 0.039, *p* = 0.008), indicating less favorable functional movement. No significant differences were detected in LE and PRO guidance. These findings suggest that DD is associated with both static and dynamic occlusal contact characteristics, particularly in the posterior region and during right lateral movement.

**Fig. 2.** Calculated occlusal contacts. Occlusal contacts, calculated using the articulation ratio (ranging from 0 to 1) proposed by the authors, were evaluated across all mandibular positions, including intercuspal position (ICP), right excursion (RE), left excursion (LE), and protrusion (PRO). Statistically significant differences (*p * < 0.05), determined by independent *t*-tests, are indicated by asterisks (*).

The study also evaluated clinical symptoms and diagnoses of the right and left TMJs in 770 patients, as illustrated in Table 4. TMJ clicking was observed in 39.4% of left TMJs and 39.1% of right TMJs, with no significant difference between sides (*p* = 0.917). Among patients with clicking, approximately 13.4% exhibited unilateral clicking, while 25.8% experienced bilateral clicking, again showing no significant side difference (*p* = 0.910). The most commonly reported types of clicking were during mouth opening (29.0%), closing (23.9%), and eccentric movements (15.6%), with no notable side-related differences (*p* = 0.998). The most prevalent disc diagnosis was disc displacement with reduction (33.0%), followed by disc displacement with reduction and intermittent locking (6.2%) and disc displacement without reduction with limited opening (2.3%), with no significant variation between the right and left TMJs. These findings indicate an absence of lateral predominance in TMJ clinical signs and diagnostic patterns.

**Table 4.** Comparison of TMJ clinical findings and diagnosis between the left and right sides in 770 patients (1540 total joints evaluated).

Variables		Right TMJ (N = 770)	Left TMJ (N = 770)	Total TMJ (N = 1540)	*p*-value
Presence of TMJ clicking^†^					
	Yes	301 (39.1%)	303 (39.4%)	604 (39.2%)	0.917
	No	469 (60.9%)	467 (60.6%)	936 (60.8%)	
Clicking site^†^					
	Unilateral	102 (13.3%)	104 (13.5%)	206 (13.4%)	0.910
	Bilateral	199 (25.8%)	199 (25.8%)	398 (25.8%)	
Types of clicking^†^					
	Opening click	219 (28.4%)	227 (29.5%)	446 (29.0%)	0.998
	Closing click	180 (23.4%)	188 (24.4%)	368 (23.9%)	
	Eccentric click	118 (15.3%)	123 (16.0%)	241 (15.6%)	
Diagnosis of articular disc^†^					
	DDwR	253 (32.9%)	255 (33.1%)	508 (33.0%)	0.954
	DDwRwIL	48 (6.2%)	48 (6.2%)	96 (6.2%)	
	DDwoRwLO	17 (2.2%)	19 (2.5%)	36 (2.3%)	
	DDwoRwoLO	0 (0.0%)	0 (0.0%)	0 (0.0%)	

*^†^Chi-square test. TMJ, Temporomandibular joint; DDwR, Disc displacement with reduction; DDwRwIL, Disc displacement with reduction with intermittent locking; DDwoRwLO, Disc displacement without reduction with limited opening; DDwoRwoLO, Disc displacement without reduction without limited opening.*

Selected factors that significantly influenced DD were further analyzed across the various disc condition groups (Table 5 and Fig. 3). Statistically significant differences were observed in age, age distribution, maximum mouth opening, mouth opening patterns, and sex (*p * < 0.05). Individuals with DD, particularly those with intermittent locking or limited opening, tended to be younger (Fig. 3A), predominantly within the 21–40 age group (Fig. 3B), and more frequently female (Fig. 3E). Maximum mouth opening measurements (pain-free, unassisted, and assisted) showed a progressive decline with increasing severity of DD, with the lowest values observed in the DDwoRwLO group (Fig. 3C). Additionally, mouth opening patterns shifted from straight to corrected or uncorrected as severity increased (Fig. 3D). These findings indicate that more advanced stages of temporomandibular disc disorders are associated with younger age, female predominance, and substantially reduced jaw mobility.

**Table 5.** Comparison of selected demographic and clinical characteristics across disc condition groups, diagnosed using clinical DC/TMD only (without MRI confirmation).

Variables		Normal Disc (N = 350)	DDwR (N = 340)	DDwRwIL (N = 59)	DDwoRwLO (N = 21)	*p*-value
Age^¶^ (yr)						
	Mean ± SD	42.7 ± 16.7^A^	39.8 ± 15.9^A,B^	34.3 ± 16.0^B^	39.0 ± 17.3^A,B^	0.001*
	[95% CI]	[41.0–44.5]	[38.1–41.5]	[30.2–38.5]	[31.1–46.9]	
Age groups^†^ (yr)						
	0–20	30 (8.6%)	26 (7.6%)	9 (15.3%)	2 (9.5%)	0.010*
	21–40	129 (36.9%)^a^	168 (49.4%)^b^	33 (55.9%)^b^	9 (42.9%)^a,b^	
	41–60	131 (37.4%)^a^	97 (28.5%)^a,b^	11 (18.6%)^b^	6 (28.6%)^a,b^	
	61–80	60 (17.1%)	49 (14.4%)	6 (10.2%)	4 (19.0%)	
Sex^†^						
	Female	208 (59.4%)^a^	234 (68.8%)^a^	51 (86.4%)^b^	18 (85.7%)^b^	<0.001*
	Male	142 (40.6%)^a^	106 (31.2%)^a^	8 (13.6%)^b^	3 (14.3%)^b^	
Previous orthodontic treatment^†^						
	Yes	71 (20.3%)	93 (27.4%)	19 (32.2%)	7 (33.3%)	0.055
	No	279 (79.7%)	247 (72.6%)	40 (67.8%)	14 (66.7%)	
Resting chin on hand^†^						
	Yes	73 (20.9%)^a^	107 (31.5%)^b^	21 (35.6%)^b^	6 (28.6%)^a,b^	0.006*
	No	277 (79.1%)^a^	233 (68.5%)^b^	38 (64.4%)^b^	15 (71.4%)^a,b^	
Patterns of mouth opening^†^						
	Straight	240 (68.6%)^a^	170 (50.0%)^a,b^	22 (37.3%)^b^	9 (42.9%)^a,b^	<0.001*
	Corrected	76 (21.7%)^a^	128 (37.6%)^b^	24 (40.7%)^b^	5 (23.8%)^a,b^	
	Uncorrected	34 (9.7%)^a^	42 (12.4%)^a,b^	13 (22.0%)^b,c^	7 (33.3%)^c^	
Maximum mouth opening^¶^ (mm)						
	Pain-free	41.8 ± 7.9^A^	40.2 ± 8.1^B^	34.0 ± 9.3^C^	26.0 ± 5.9^D^	<0.001*
	Unassisted	45.2 ± 6.5^A^	45.6 ± 6.8^A^	40.1 ± 8.2^B^	30.8 ± 5.2^C^	<0.001*
	Assisted	47.9 ± 6.1^A^	48.8 ± 6.4^A^	44.7 ± 7.5^B^	34.4 ± 5.9^C^	<0.001*

^†^Chi-square test; ^¶^one-way ANOVA test; *statistical significance (p < 0.05). ^A,B,C,D^statistical significance from the Tukey’s post hoc test; ^a,b,c^statistical significance from the compare proportions test. DDwR, Disc displacement with reduction; DDwRwIL, Disc displacement with reduction with intermittent locking; DDwoRwLO, Disc displacement without reduction with limited opening.

**Fig. 3.** Demographic and clinical comparisons across disc condition groups: Normal Disc, DDwR, DDwRwIL, and DDwoRwLO. (A) Age: Younger age was associated with more severe disc conditions (*p* = 0.001). (B) Age Group: The 21–40 age group was most common in DD cases (*p* = 0.010). (C) Maximum Mouth Opening: All measures decreased with severity, lowest in DDwoRwLO (*p *< 0.001). (D) Mouth Opening Patterns: More severe cases showed increased corrected or uncorrected deviation of mouth opening (*p * < 0.001). (E) Sex: Female predominance increased with DD severity (*p * < 0.001). Different capital letters indicate significant differences based on Tukey’s *post hoc* test; lowercase letters indicate significant differences from compare proportion tests. DDwR, Disc displacement with reduction; DDwRwIL, Disc displacement with reduction with intermittent locking; DDwoRwLO, Disc displacement without reduction with limited opening.

## 4. Discussion

This retrospective study investigated the associations between various demographic, behavioral, clinical, and occlusal factors and articular DD among 770 Thai patients (1540 joints). The findings provide meaningful insights into the characteristics of TMD, particularly DD. Several demographic and clinical factors were found to be associated with articular DD. These results reject the null hypothesis that there was no significant association between demographic or clinical characteristics and the presence of articular DD in Thai TMD patients.

A key finding was the significant association between DD and younger age, with the condition being more prevalent among patients aged 21–40 years. This supports earlier studies that suggest DD often develops during early adulthood [20, 21], potentially due to the cumulative effects of mechanical stress, oral habits, or hormonal influences [5, 22]. Additionally, female patients were significantly more likely to experience DD [21], aligning with a well-established body of evidence indicating a higher prevalence of TMDs among women [3]. Hormonal, anatomical, and psychosocial factors may contribute to this sex-based predisposition [23].

Among behavioral factors, a history of orthodontic treatment and the habit of resting the chin on the hand were significantly associated with DD. While orthodontic treatment has been debated as a risk factor for TMD [24, 25], the present study suggests a potential link, possibly due to alterations in occlusion or mandibular positioning [26]. The chin-resting habit may place asymmetrical loading on the TMJ, leading to biomechanical stress and eventual DD [27]. Although unilateral chewing and sleeping on one side may affect TMJs, causing slight posterolateral movement of the ipsilateral condyle and anteromedial displacement of the contralateral condyle [28, 29], these behaviors were not significantly associated with DD in this study. Additionally, poor posture at work was not associated with DD, which aligns with the findings of Rocha *et al*. [30], who reported no significant relationship between body posture and DD. This suggests that not all repetitive or asymmetrical habits exert the same influence on TMJ health.

Interestingly, general occlusal characteristics, such as overjet, overbite, midline deviation, and occlusal scheme, were not significantly associated with DD, suggesting that static occlusal relationships may not play a major role in the development of disc pathology. Although many previous studies have reported an association between malocclusion and DD [31, 32], those studies often did not compare malocclusion in patients with DD against those with normal disc positions [33]. Previous studies may have lacked control groups with normal disc positions, limiting the ability to discern whether malocclusions are truly predictive or merely co-existing conditions. While the role of occlusal characteristics in the etiology of DD remains debated [9], the findings of this study suggest that such features are not significantly associated with the presence of DD.

Occlusal contact analysis provided additional insights into the relationship between the number of occlusal contacts and DD. These parameters were assessed using a specialized research tool developed by the authors. As shown in Fig. 2, patients with DD exhibited significantly lower posterior static articulation and total static articulation ratios, indicating reduced occlusal stability. This observation indicates that insufficient posterior occlusal support may be a contributing factor to the development of DD. Furthermore, lateral excursion patterns also appeared to influence the incidence of DD, particularly when occlusal contact on the non-working side was increased during mandibular movement. A notable asymmetry was observed between right and left excursions. During the right excursion, there was a significant reduction in the working-side occlusal contacts (*p* = 0.015) accompanied by increased contacts on the non-working side (*p* = 0.008). In contrast, the left excursion maintained relatively high occlusal contact on the working side, with no statistically significant difference observed (*p* = 0.973). Although previous studies have reported an association between non-working interferences and TMD [34, 35], these results suggest that a combination of decreased working-side contact and increased non-working-side contact may be critical factors in the development of DD. Accordingly, clinicians should evaluate occlusal contact patterns on both working and non-working sides during functional jaw movements when assessing the risk or presence of DD.

DD during right lateral jaw movement, characterized by decreased working contacts and increased non-working contacts, may result from several biomechanical factors [36]. Occlusal interferences and the absence of proper protective guidance, such as canine guidance, increase lateral joint forces, thereby overloading discal attachments [37]. Muscle imbalances, particularly of the lateral pterygoid, can further disrupt disc-condyle coordination [38]. Altered condylar loading generates shear and tensile stresses that predispose the disc to anterior or anteromedial displacement [39, 40]. Repeated loading may stretch or fatigue ligaments, reducing disc stability, while joint morphology, such as a steep articular eminence or shallow glenoid fossa, can alter condylar paths and increase the risk of displacement [41].

Functional assessments revealed that patients with DD were significantly more likely to exhibit either corrected or uncorrected mandibular deviation during mouth opening (Fig. 3D). The diagnostic criterion for uncorrected deviation is defined as a mandibular deviation exceeding 2 mm [42]. While this threshold is commonly used, the present findings suggest that it may not reliably differentiate between specific DD subtypes, as statistically significant differences were observed among groups but without clear diagnostic separation. This implies a need to revisit and refine the current deviation criteria for improved diagnostic specificity.

Additionally, the widely accepted diagnostic threshold for maximum mouth opening, 40 mm, is often used to distinguish between DDwR and DDwoR [7]. Data from Fig. 3C support the applicability of this criterion to the Thai population. Patients with normal disc positions and those with DDwR typically demonstrated maximum mouth openings greater than 40 mm, indicating no significant limitation. In contrast, patients diagnosed with DDwRwIL and DDwoRwLO exhibited mouth opening capacities below 40 mm. To further differentiate between DDwRwIL and DDwoRwLO, clinical recommendations emphasize evaluating both unassisted opening (maximum voluntary opening despite discomfort) and assisted opening (examiner-facilitated mandibular stretching using finger placement on anterior teeth). These two approaches, when applied with the 40-mm cutoff, may enhance the diagnostic distinction between DDwRwIL and DDwoRwLO.

The findings of this study highlight the importance of incorporating dynamic occlusal assessments into routine TMD evaluations. For general dentists, who are often the first point of contact for patients with TMD-related complaints, recognizing functional occlusal patterns, such as reduced posterior support or non-working side interferences, may facilitate the early detection of patients at risk for DD. Basic clinical tools, such as articulating paper or shim stock, can be used to evaluate excursive contacts [43]. Patients presenting with reduced posterior occlusal contact, diminished working-side contact, or increased non-working contacts should be considered at risk for articular DD. Furthermore, in complex cases involving additional factors, such as systemic disease, neuromuscular disorders, or psychological conditions, referral to a specialist is recommended. For prosthodontists, who manage complex occlusal rehabilitation and TMJ function, these findings reinforce the clinical utility of evaluating articulation ratios and lateral guidance schemes [44]. DD appears more closely associated with functional occlusal discrepancies than with traditional static parameters like overjet or overbite. Incorporating dynamic occlusal metrics into prosthodontic treatment planning could improve diagnostic precision and facilitate more accurate occlusal modifications, ultimately improving long-term joint stability and patient outcomes.

This study had several limitations. First, the diagnostic criteria were based on the DC/TMD protocol, which relies on patient history and clinical examination and is widely recognized for its high validity. However, imaging techniques, such as magnetic resonance imaging (MRI), were not employed, which may have limited the diagnostic accuracy, particularly in detecting subtle or asymptomatic DD [45]. Future studies incorporating imaging modalities could provide more definitive diagnoses and further validate the observed associations [46]. Additionally, the potential correlation between DD and degenerative joint changes warrants further investigation [47]. Second, the study population was drawn from the Occlusion and Orofacial Pain Clinic, which primarily serves patients with TMD and related conditions. As a result, the frequency of DD in this sample was relatively high (420 of 770 cases, or 54.5%). By contrast, a previous report from the same dental hospital found that only 6.1% of the general dental patient population was diagnosed with TMD [4]. To approximate the prevalence of DD in the broader dental population, this proportion can be multiplied by the prevalence of DD among TMD patients: 6.1% × 54.5% = 3.32%. On a global scale, where the prevalence of TMDs has been reported as 29.5% [48], the estimated prevalence of DD would be 29.5% × 54.5% = 16.1%. These indirect estimates suggest that the high frequency observed in our study reflects the specialized clinical setting, and caution is needed when generalizing the findings to wider populations. Third, occlusion in this study was assessed by dentists while patients were lying in a supine position on the dental unit, which differs from the natural upright head position. Both static and dynamic occlusion may, therefore, be altered compared with normal function. This positional difference could affect jaw function and dental occlusion. Future studies should confirm these findings in the upright position to minimize the influence of mandibular retrusion.

## 5. Conclusions

This study highlights several significant associations between demographic, behavioral, clinical, and occlusal factors and articular DD in Thai patients. Younger age, female sex, a history of orthodontic treatment, and the habit of resting the chin on the hand were significantly associated with DD. Although DD was not strongly associated with general occlusal characteristics, an association was found with reduced posterior occlusal support and altered functional contact patterns. Additionally, limitations in mouth opening and mandibular deviation were helpful in distinguishing between DD subtypes. These results underscore the multifactorial nature of DD and emphasize the importance of comprehensive clinical and functional assessment in diagnosis and management.
